# A novel DNA profiling method for individual sample matching and its applications to wildlife diagnostics

**DOI:** 10.1038/s44185-026-00148-3

**Published:** 2026-07-09

**Authors:** Kyle M. Ewart, Rob Ogden

**Affiliations:** 1 Royal (Dick) School of Veterinary Studies and the Roslin Institute, University of Edinburgh, Edinburgh, UK; 2 TRACE Wildlife Forensics Network, Edinburgh, UK

**Keywords:** Biological techniques, Ecology, Ecology, Genetics, Plant sciences

## Abstract

The use of DNA analysis to match individual biological samples is central to many wildlife diagnostic applications and is particularly valuable in illegal wildlife trade investigations. However, although thousands of wildlife and plant species are affected by illegal trade, DNA profiling systems for individual identification exist for only a small number of taxa. This is primarily due to the need for extensive population reference data to enable conclusive interpretation of DNA profile matches, a key requirement in diagnostic contexts. Here, we introduce a novel approach that does not require population reference data to interpret an individual profile match and can be used with any set of genetic markers on a case-by-case basis. This approach, called ‘heterozygote analysis for reference-free probability assessment’ (*HARP*), is based on ultra-conservative match probability estimates using only heterozygote genotypes and assuming both alleles have a frequency of 0.5 in the population. *HARP* effectively eliminates the need for population reference data and standard marker panels and is therefore completely adaptable to any novel individual identification scenario. We demonstrate *HARP* using three case studies spanning a range of species and diagnostic scenarios.

## Introduction

The use of DNA analysis to match individual biological samples underpins many diagnostic applications and is routinely utilised in forensic science^1–3^, authenticity testing^4,5^ and conservation genetics^6–8^. A key application of DNA-based individual identification is detecting, monitoring and investigating the illegal wildlife trade. Wildlife trafficking is a major driver of biodiversity loss, affecting thousands of animal and plant species worldwide^9,10^. Many wildlife trade investigations hinge on establishing whether biological samples originate from the same individual organism or from different individuals. For example, authorities may need to determine whether a seized wildlife part (e.g. a horn or skin) originated from a particular carcass, whether biological material on a suspect (e.g. blood) derives from a poached animal, or whether products encountered at different stages of a supply chain correspond to the same source (e.g. linking timber to a tree stump).

In addition to generating DNA profiles through genotyping samples using a panel of genetic markers, a conclusive and justifiable interpretation of any resultant DNA profile match is essential. Two samples with identical DNA profiles do not necessarily derive from the same individual; different individuals can share the same profile by chance, particularly when only a limited number of genetic markers are examined. Accordingly, in diagnostic contexts, individual identity cannot be inferred from a DNA profile match alone but must also incorporate an assessment of the strength of the match given the markers’ characteristics and the observed profile. This typically involves estimating the probability of observing the profile in the general population, known as the genotype match probability. Estimating this probability has received significant statistical refinement over the past forty years^11^, but has always required large-scale population genetic data for the specific DNA profiling system in use, creating a significant barrier to the development of novel DNA profiling systems.

A DNA profile database generated from population-wide reference samples is required to estimate the associated loci allele frequencies, which underpin match probability calculations^12^. The number of samples required to obtain accurate and precise allele frequency estimates depends on numerous factors (e.g. the distribution of genetic diversity and the biology of the target species), though generally more than 30 samples are required from the target population^13–15^. Collecting reference samples poses numerous practical and ethical challenges, particularly for wide-ranging, rare, or dangerous species, or for those requiring special permits. Hence, the construction of such a database incurs considerable research and development costs for each species.

Even when reference samples are available, the design and development of a taxon-specific marker panel requires considerable resources, which is often not economically viable for species that are not tested in large numbers. Further, transferring an established DNA profiling system and associated population reference data to a new laboratory introduces additional challenges; the reference data may be unsuitable if the target population changes, and the system may require calibration across laboratories (e.g. using standard genotyping samples) to ensure compatibility^16^. Consequently, DNA-based individual identification is largely restricted to abundant model species (e.g. humans and domestic animals) or those expected to undergo routine testing, and is generally infeasible for rarer, less-studied taxa. These limitations are particularly evident in wildlife trade applications, where most trafficked species lack established DNA profiling systems and population reference datasets, and many laboratories have no access to the few systems that do exist.

Here, we present a novel approach to DNA profiling that does not require population genetic data or any specific marker panel to interpret a profile match, hence can be applied to any species, and as a general method, is easily transferable across laboratories. Traditional profiling systems comprise relatively few independent genetic markers (typically < 25), with ample power to differentiate individuals due to the rarity of the genotype observed at each marker. However, it is now routine to generate hundreds to thousands of genome-wide markers from a DNA sample. We build on this capability and propose an updated approach for estimating match probabilities, which we term ‘heterozygote analysis for reference-free probability assessment (*HARP*)’. *HARP* considers only heterozygous site matches and applies ultra-conservative assumptions: that each marker is biallelic, with alleles present at equal frequencies in the population (i.e. *p* = *q* = 0.5). The resulting match probabilities are generated without the need for specific marker panels or associated population reference data. We demonstrate the utility of *HARP* using three case studies. The adaptability of *HARP* across species and use cases has the potential to transform the use of individual DNA profiling in wildlife diagnostics.

## Results

### HARP assumptions and discriminatory power

*HARP* match probabilities are calculated without population reference data by only considering heterozygous loci (see ‘Methods’ for the full derivation). *HARP* assumes two alleles per heterozygote locus, both with a population frequency of 0.5. This is conservative: when allele frequencies differ from 0.5, or when more than two alleles exist in the population, match probabilities decrease (i.e. become more powerful).

*HARP* is underpinned by two additional key assumptions. First, markers are assumed to be in linkage disequilibrium (LD) to justify the calculation of cumulative probabilities across independent loci. This assumption can be met by filtering markers based on genomic location or by carrying out LD testing. Second, markers are assumed to be in Hardy–Weinberg equilibrium (HWE). A conventional approach to account for HWE deviations when calculating match probabilities is to incorporate a theta (*θ*) correction^11^, which addresses heterozygote deficiency (e.g. as a result of population structure or individual relatedness). However, incorporating *θ* > 0 into *HARP* decreases the resultant match probability; hence, we conservatively assume *θ* = 0. A less common issue, but one that may lead to underestimation of *HARP* match probabilities, is heterozygote excess. This can arise through selection scenarios such as heterozygote advantage, deleterious recessive alleles, advantageous dominant alleles and disassortative mating (elaborated in the Supplementary Information). These scenarios are accounted for by incorporating a correction factor, *s*, representing the proportion of the markers affected by scenarios leading to heterozygote excess.

Applying *HARP* to a single heterozygous marker, conservatively assuming *s* = 0.1 (see Supplementary Information for justification), results in a match probability of ~0.536; i.e. there is a 53.6% chance of observing this same heterozygote genotype in the general population. This can also be expressed as a likelihood ratio (LR): when matching a sample to a suspected donor, observing this genotype is 1.866 times more likely if the sample derives from the donor rather than from an unrelated individual. *HARP* probabilities can be multiplied across multiple heterozygous loci provided they segregate independently. This results in exponentially decreasing match probabilities, yielding considerable discriminatory power from relatively few markers, despite the conservative assumptions underpinning the calculation. The number of markers required and the associated power of *HARP* depend on the application and species in question. For example, for 25 independent heterozygote markers, assuming *s* = 0.1, the probability that another individual in the population shares this heterozygote marker profile is <1.7 × 10^−7^, equivalent to an LR of >5.9 million, providing ample power for many wildlife forensic applications. In a wildlife forensic context, for a genuine individual match, generally the LR should be considerably higher than the target total population size.

### Case studies

**Fig. 1:**
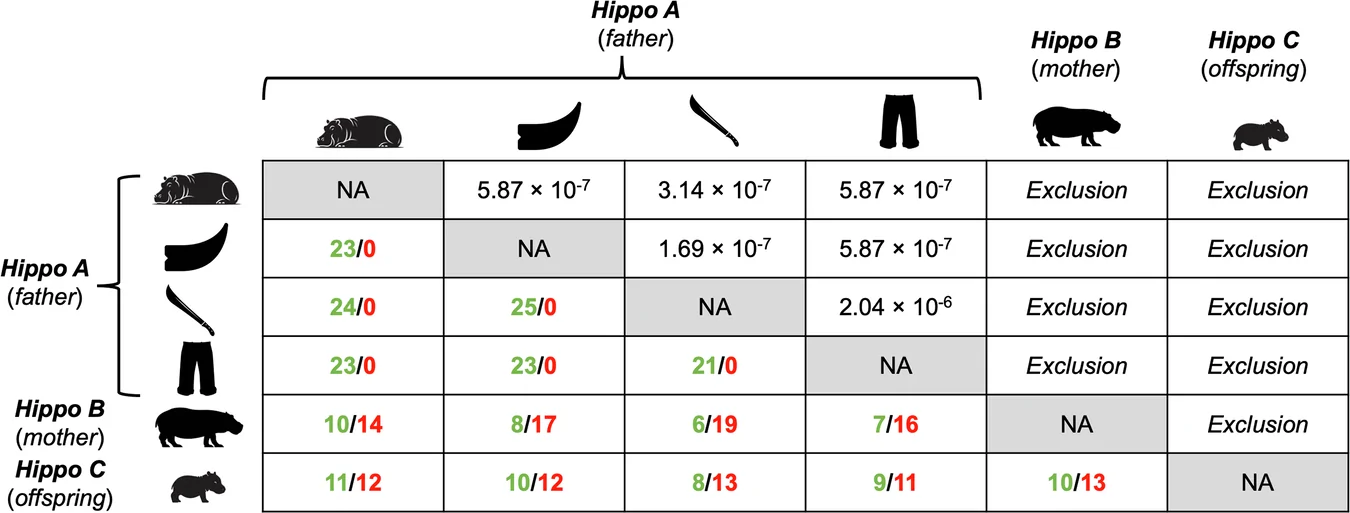
The number of matches (green) and mismatches (red) between samples in the hypothetical hippopotamus poaching case study (lower diagonal) and their associated match probabilities (upper diagonal). For hippo A, four independent datasets were used to simulate results from different evidence items (hippo carcass, hippo ivory and trace evidence on weapons and clothing). We assumed *s* = 0.1 for the *HARP* match probability calculation.

We applied *HARP* to a hypothetical hippopotamus (*Hippopotamus amphibius*) poaching case, in which a carcass is discovered, a hippo tooth (ivory) is recovered from a vehicle, and suspected poachers are subsequently detained in possession of a knife and clothing, displaying traces of blood (Fig. 1). Heterozygote profiles from the four hippopotamus whole genome datasets generated from the same individual (using independent library preparations; Supplementary Table S1) matched across 21-25 heterozygote single nucleotide polymorphism markers (SNPs), with no mismatches, yielding match probabilities ranging from 2.04 × 10^−6^ to 1.69 × 10^−7^ (Fig. 1). Profiles from different individuals, including first-degree relatives, were successfully excluded. This case study demonstrates the application of *HARP* when no marker panel is available. Notably, we took stringent measures to ensure markers were in LD by only analysing a single SNP per scaffold of the hippopotamus genome (for scaffolds >1 Mb; see ‘Methods’).

We also applied *HARP* to two species with established SNP profiling systems, but analysing the datasets without using the associated population reference data. A 144 SNP panel for snow leopards (*Panthera uncia*)^8^ yielded an expected 15 heterozygotes between matching samples (see Supplementary Information), corresponding to a *HARP* match probability of 8.63 × 10^−5^, and a 128 SNP panel for bigleaf maple (*Acer macrophyllum*)^17^ yielded an expected 40 heterozygotes between matching samples, corresponding to a *HARP* match probability of 1.46 × 10^−11^ (Table 1). These examples represent applications to conservation genetic monitoring and timber trade authenticity testing, respectively.

**Table 1:** Expected number of heterozygote matches for the snow leopard and bigleaf maple case studies and their associated match probabilities

Case study species	Marker panel	Non-missingness proportion	Observed heterozygosity	Expected number of matching heterozygotes	Match probability
Snow leopard	144 SNP panel genotyped using high-throughput amplicon sequencing	57%	0.32	15	8.63 × 10^−5^
Bigleaf maple	128 SNP panel genotyped using a MassARRAY® iPLEX™ system	95%	0.344	40	1.46 × 10^−11^

The number of heterozygotes for each case study is based on the missingness and observed heterozygosity reported for the associated profiling systems (see Supplementary Information). We assumed *s* = 0.1 for the *HARP* match probability calculations.

## Discussion

Although DNA-based individual identification is widely applicable across wildlife diagnostic contexts, it has been implemented for only a small number of species. This is primarily due to the substantial research and development required to develop species-specific marker panels and associated population reference data. *HARP* addresses this barrier by enabling individual identification analysis without the need for these prerequisites, and can therefore be applied to any species for which DNA profiling systems have not been developed.

*HARP* will be particularly beneficial in wildlife forensic applications, as illustrated in the hippopotamus poaching case study. A lack of testing capacity likely hinders prosecution in many cases that hinge on matching samples. Unlike conventional forensic DNA profiling methods based on specific panels of short tandem repeats (i.e. STRs^12^) or SNPs^18^, which require marker validation, the whole genome approach used in the hippopotamus case study does not explicitly characterise individual markers. While this may raise instinctive concerns within the forensic genetics community, we believe that any associated risks can be effectively mitigated through stringent and extremely conservative bioinformatic pipelines that only retain highly reliable SNPs, minimise LD, and correct for markers with potential excess heterozygosity through the use of a conservative value for *s*. Although the analysis of whole genome data carried out for the hippopotamus case study was conservative, the approach has not been validated for forensic use (given the relatively small scale of the case study). We encourage further discussions and research into how genomic approaches and the application of *HARP* can be validated for forensic individualisation analyses.

Beyond forensics, *HARP* is broadly applicable to any diagnostic context requiring individualisation and could transform how DNA-based diagnostic systems are designed and managed for product traceability. Audit systems conducting one-up-one-down supply chain verification for any species, but particularly those with high-value individuals, could apply this method without prior species-specific research and development. For example, *HARP* could be applied to match logs to tree stumps in illegal logging compliance testing involving multiple timber species, where individual trees are harvested and labelled within a concession.

*HARP* is agnostic to how heterozygote markers are generated. When existing marker panels are used to generate heterozygote profiles, no inter-laboratory calibration is required (often necessary to enable shared population reference datasets), facilitating straightforward transfer of profiling systems across laboratories. A whole genome sequencing approach, as illustrated in the hippopotamus case study, does not require a specific marker panel and could be applied to any species. A relatively high-quality reference genome was available for hippopotamus, which enabled us to confidently call SNPs, minimise false heterozygote calls, and evaluate linkage disequilibrium (LD); such genome resources are increasingly available across a growing number of species through global reference sequencing initiatives (e.g. DToL, ERGA, EarthBioGenome). If no reference genome is available for the species in question, a closely related species’ genome could potentially be used to account for LD, though greater care would be required to ensure accurate LD estimates and genotype calls. We used very conservative LD measures (i.e. retained a single SNP near the midpoint of the longest scaffolds; see ‘Methods’). Thousands of genome-wide SNPs could potentially be utilised, dramatically increasing discriminatory power, particularly if LD decay is known for the target population, or if linkage is explicitly accounted for in the match statistics^19–21^. However, increasing the number of markers compared may increase the risk of false mismatches (cases where a true match is incorrectly excluded), though these can be managed using validated error rate thresholds.

The per-sample sequencing effort and associated costs required for this *HARP* whole genome approach depend on genome size and sequence quality. Costs could be substantially reduced through enrichment or genome complexity reduction methods (e.g refs. ^22–24^). We utilised relatively high coverage datasets (~10× coverage), with stringent thresholds to ensure highly reliable SNP calls. However, lower coverage data could potentially be used, provided the associated increase in error rate is accounted for or integrated into the match statistics (e.g ref. ^25^).

In summary, *HARP* has the potential to revolutionise DNA profiling for matching biological samples. By eliminating the need for species-specific development and population reference data, *HARP* paves the way for a dramatic increase in novel DNA profiling applications across forensic science, diagnostics, and research.

## Methods

### HARP approach

Here we derive and justify a per-marker match probability without the need for a population genetic reference database. At each step, we take a conservative approach for this derivation.

Assuming two alleles (A and B) occur at a given marker, the maximum probability, *P*, of observing a heterozygous (AB) genotype is *P*(AB) = 0.5. The corresponding maximum probabilities for AA and BB genotypes are *P*(AA) = *P*(BB) = 1, making homozygous loci uninformative in the absence of population data. While a conservative heterozygote genotype probability of *P*(AB) = 0.5 is less powerful than those typically calculated using population reference data (i.e. if *p* or *q* is <0.5, or if there are >2 alleles), when using a relatively large number of markers in combination, it is possible to recover sufficient heterozygote genotype matches to produce extremely small match probabilities.

*HARP* assumes HWE, which is violated through either deficiency or excess in observed heterozygote genotypes. Only heterozygote excess is relevant to *HARP* and is accounted for by estimating the proportion of markers affected and introducing an associated correction factor, *s*, as follows:

1 $$P\,\left({AB}\right)={0.5}^{\left(n\times \left(1-s\right)\right)}$$

where *n* is the number of DNA markers with matching heterozygous genotypes in the profile (i.e. where both samples being compared have an identical heterozygote genotype). This equation holds even when comparing highly related individuals (e.g. first-degree relatives).

*HARP* also assumes markers are not linked, which is addressed by filtering markers to minimise LD. See the Supplementary Information for further details of the *HARP* assumptions and corrections.

### Case study 1: hippopotamus whole genome sequencing

*HARP* was applied to a hypothetical hippopotamus poaching case using whole genome sequencing datasets, providing a large set of heterozygous markers available for analyses. DNA profile comparisons were made between samples from hippopotamus ivory, trace evidence on the knife and clothing, the carcass, and two other hippopotamus individuals (Fig. 1). The ivory, trace evidence and carcass were derived from the same individual, while the two additional individuals formed a trio (sire, dam and offspring) to assess the discriminatory power of the approach among closely related individuals. Whole genome sequencing data were available for the hippopotamus samples involved in this hypothetical case. This case study was chosen for two key reasons: (1) it represents a realistic wildlife forensic application of *HARP*; (2) it involves a species for which no marker panel or associated population reference data are available.

Each of the six evidence samples from this hypothetical hippopotamus poaching case was represented by an independently re-sequenced hippo genome (Supplementary Table S1), obtained from two studies^26,27^; i.e. four whole genome Illumina datasets were generated from different libraries of the same individual hippo (GenBank Accession numbers: SRR5663646, SRR5663647 and SRR5663649 and SRR17072459), while two datasets completed the trio (GenBank Accession numbers: SRR17072457 and SRR17072472). All datasets were produced with Illumina 100 bp PE sequencing (insert sizes varied between datasets).

We carried out pairwise comparisons of SNPs between each of the six datasets. The step numbers below correspond to the pipeline described on GitHub (https://github.com/KyleEwart/HARP_WGS_pipeline):

- *Step 1. Subsampling raw reads:* To standardise the variant calling and subsequent comparisons, we randomly downsampled each paired raw sequencing dataset to ~10× coverage (based on a 3.1 Gb genome size) using seqtk v1.4 (https://github.com/lh3/seqtk). This resulted in 310 million reads per sample (which includes both R1 and R2 reads).
- *Step 2. Trimming reads:* Reads were trimmed for quality, and adaptors were removed using the BBDuk tool in BBMap v38.84^28^.
- *Step 3. Aligning reads to genome:* The trimmed reads were mapped to a hippo genome assembly (GCA_030028035.1; Vertebrate Genomes Project^29^) with bwa mem v0.7.18^30^. PCR duplicates, multi-mappers, and reads with ≥15 bp of soft-clipped sequence (representing potential alignment artefacts) were removed using samtools v1.20^31^, and sequencing depth was assessed using mosdepth v0.3.3^32^.
- *Step 4. SNP calling:* SNPs were called using FreeBayes v1.3.2^33^, implementing a minimum coverage of 8 and a minimum alternate allele fraction set of 0.1. For each alignment, FreeBayes was used to generate both a VCF and a gVCF for subsequent comparisons.
- *Step 5. Characterising repeat regions:* We utilised RepeatModeler v2.0.1^34^ and RepeatMasker v4.0.6^35^ to generate a repeat database and produce a BED file specifying repeat regions. This was used for SNP filtering applied in ‘*Step 6*’.
- *Step 6. Comparing heterozygotes across the VCF and gVCF files:* The VCF and gVCF outputs were compared between samples. Both the VCF and gVCF files underwent stringent filtering using bcftools v1.11^31^: only biallelic SNPs were retained, and required a quality score >30, depth ≥12, alternate allele frequency >0.3, and mapping quality ≥40 (for both the reference and alternate alleles). We also implemented a conservative maximum coverage filter (SNPs with depth >14 were removed) to ensure false heterozygote calls caused by erroneously collapsed paralogs were excluded. Homozygote calls were removed if ≥1 read supported an alternate allele. Then, only heterozygotes were retained in the VCF, and the corresponding sites were extracted from the gVCF of the other sample being compared. We used bcftools to remove SNPs located in repeat regions (using the BED file generated in ‘*Step* 5’). To minimise the chance of linkage disequilibrium, we retained a single SNP near the midpoint of the longest scaffolds in the hippopotamus genome. To do this, we identified scaffolds >1 Mb in length (*n* = 41) and produced a BED file specifying regions 500 kb from each scaffold end (thereby ensuring SNPs were at >1 Mb distant from one another). We then utilised bcftools and an awk function to retain the SNP nearest to the midpoint of each region specified in this BED file.

This pipeline produced two VCF files per pairwise comparison, as the VCF for each sample was compared to the gVCF in all other samples, and vice versa. We retained the VCF/gVCF combination that yielded the highest number of SNP comparisons.

### Case studies 2 and 3: bigleaf maple and snow leopard profiling systems

We also applied *HARP* to two additional case studies involving species that have established DNA profiling systems: (1) a 128 SNP panel for bigleaf maple, used to assess trade authenticity of this commonly traded timber species^17^, and (2) a 144 SNP panel for snow leopards, developed for conservation genetic applications^8^ (Table 1). In these analyses, the existing systems were treated as if suitable reference datasets were unavailable (or unsuitable) in order to illustrate how *HARP* would perform under such conditions. The expected number of heterozygote matches between profiles was estimated from reported heterozygosity and missingness in the respective studies (elaborated in the Supplementary Information).

## Supplementary information

Supplementary Information

## Data Availability

Analyses carried out in this study are based on previously generated and openly accessible datasets. The relevant studies and accession numbers are available within the paper and its Supplementary Information.

## Code availability

The scripts used for hypothetical hippopotamus poaching case based on the whole genome datasets are available in GitHub: https://github.com/KyleEwart/HARP_WGS_pipeline.
