# Supplementary material for: A novel DNA profiling method for individual sample matching and its applications to wildlife diagnostics
Source: NPJ Biodivers. 2026 Jul 9;5:41. doi: 10.1038/s44185-026-00148-3 (PMC13727668; doi:10.1038/s44185-026-00148-3)
Supplement: Supplementary file 1 [file 44185_2026_148_MOESM1_ESM.docx]

Supplementary Information

**Additional details on *HARP* assumptions and corrections**

*HARP* assumes Hardy-Weinberg Equilibrium (HWE). Typically, deviation to HWE due to high coancestry between individuals (e.g. as a result of population structure or individual relatedness) is corrected for in match probability calculations using theta (*θ*) [1]. However, when only considering heterozygote matches, the use of theta is less conservative; i.e. when *θ*>0, *P*(AB) < 0.5. Hence, to be conservative, we assume *θ*=0, though *θ* can be incorporated into *HARP* if a justifiable value is available for a given study system (e.g. if assumptions of high coancestry are warranted).

Although heterozygote deficiency is a more prominent issue than heterozygote excess (hence the typical correction using *θ*), the latter can still arise. Heterozygote excess is more relevant to *HARP* as it will result in underestimating heterozygote match probabilities. Such scenarios include:

- heterozygote advantage, where heterozygote individuals have a fitness advantage over homozygotes.
- deleterious recessive alleles, causing a deficit in homozygote recessive individuals, thereby inflating the number of heterozygotes and dominant allele homozygotes in the population.
- advantageous dominant alleles, causing an increase in heterozygotes and dominant allele homozygotes in the population.
- disassortative mating, favouring unions between opposite homozygote genotypes, leading to an excess of heterozygote offspring.

Of the scenarios outlined above, purifying selection is expected to be by far the most prevalent, whereby deleterious recessive homozygotes are selectively removed from the population. Estimates of the proportion of the human genome potentially subject to purifying selection varies across studies (3-9% [2,3]). Further, these scenarios will affect specific genomic regions, not genome-wide heterozygosity. Since it is not possible to test for HWE deviations on a per-marker basis in the absence of population genetic data, for the case studies we conservatively assume that said selection scenarios affect 10% of markers (i.e. *s*=0.1). Where marker information is available, and the loci have been shown to be unaffected by such selection, *s*=0 could be applied instead. A false heterozygote error rate (resulting in false matches) could also be incorporated into *s*.

*HARP* also assumes linkage disequilibrium (LD) to justify calculation of cumulative probabilities across independent markers. A possible extension to *HARP* could retain dependent markers and explicitly incorporate linkage information (e.g. [4]). However, for the applications we present here, markers were filtered appropriately to minimise the chance of LD; consequently, some information on marker locality and/or LD testing is required, which can be obtained from a genome assembly (e.g. see the hippopotamus poaching case study).

**Expected number of matching heterozygotes for snow leopard and bigleaf maple**

We calculated the expected number of matching heterozygotes for two existing DNA profiling systems for snow leopard [5] and bigleaf maple [6] based on reported heterozygosity and the proportion of non-missingness of the marker panels. Non-missingness here refers to the proportion of loci of the complete marker panel; the overlap between two profiles across their non-missing portions dictates how many markers are compared. For the 144 SNP panel for snow leopards, published by Solari *et al.* [5], we used an observed heterozygosity of 0.32 (from Figure 2F in [5]) and a non-missingness proportion of 0.57. Solari *et al*. [5] ran multiple replicates per sample, therefore we based our non-missingness estimate on the replicate with the highest number of genotyped SNPs (from Table S3 in [5]), assuming that profiles were not merged across replicates. To be conservative, we further assumed that missingness was randomly distributed across loci within each profiling run. Therefore:

$$Hypothetical number of overlapping SNPs between profiles=0.57 \times0.57 \times144=46.79$$

Here, ‘overlapping’ is referring to genotyped markers common to two independently produced individual profiles. This is a ‘hypothetical’ value as the DNA profiles weren’t actually compared. The number of expected ‘matching’ overlapping heterozygote sites is then based on the missingness and heterozygosity of the profiles. Hence:

$$Expected number of matching heterozygotes=0.32 \times46.79=\sim15$$

For the 131-locus panel for bigleaf maple, published by Dormontt *et al.* [6], we excluded the 3 indel markers (therefore retained 128 SNPs), and used an observed heterozygosity of 0.344 (based on Table S3 in [6]) and a non-missingness proportion of 95%. As Dormontt *et al.* [6] employed a minimum locus-pass rate of 95%, we conservatively assumed that the two profiles being compared were at this minimum call rate. Therefore:

$$Hypothetical number of overlapping SNPs between profiles=0.95 \times0.95 \times128=115.52$$

Hence:

$$Expected number of matching heterozygotes=0.344 \times115.52=\sim40$$

**Supplementary Information Tables**

**Supplementary Table S1.** Details on the datasets used in the hippopotamus hypothetical poaching case study. During our analyses, we inadvertently identified that one of the hippos in the Bergeron *et al*. [7] trio (SRR17072459) was the same individual represented by the three ‘replicate’ datasets from Árnason *et al.* [8] (confirmed by a co-author who had contributed samples to both studies).

| **GenBank Accession** | **Source** | **Individual in hypothetical case** |
| --- | --- | --- |
| SRR5663646 | Árnason *et al*. [8] | Hippo A - carcass |
| SRR5663647 | Árnason *et al*. [8] | Hippo A – canine |
| SRR5663649 | Árnason *et al*. [8] | Hippo A – blood spot on machete |
| SRR17072459 | Bergeron *et al*. [7] | Hippo A - blood spot on clothing |
| SRR17072457 | Bergeron *et al*. [7] | Hippo B |
| SRR17072472 | Bergeron *et al*. [7] | Hippo C |

**Supplementary Information References**

1. National Research Council Ii, National Research Council Committee on DNA Forensic Science, The Evaluation of Forensic DNA Evidence. 1996, Washington, D.C.: National Academy Press.
2. Lindblad-Toh, K., et al. A high-resolution map of human evolutionary constraint using 29 mammals. *Nature* **478,** 476-482 (2011).
3. Rands, C. M., Meader, S. Ponting, C. P. & Lunter, G. 8.2% of the human genome is constrained: variation in rates of turnover across functional element classes in the human lineage. *PLoS Genet.* **10,** e1004525 (2014).
4. Vohr, S. H., Buen Abad Najar, C. F., Shapiro, B. & Green, R. E. A method for positive forensic identification of samples from extremely low-coverage sequence data. *BMC Genom.* **16,** 1034 (2015).
5. Solari, K. A., et al. Next‐generation snow leopard population assessment tool: multiplex‐PCR SNP panel for individual identification from faeces. *Mol. Ecol. Resour.* **24,** e14074 (2024).
6. Dormontt, E. E., et al. Forensic validation of a SNP and INDEL panel for individualisation of timber from bigleaf maple (*Acer macrophyllum* Pursch). *Forensic Sci. Int. Genet.* **46,** 102252 (2020).
7. Bergeron, L. A. et al. Evolution of the germline mutation rate across vertebrates. *Nature* **615,** 285-291 (2023).
8. Árnason, Ú., Lammers, F., Kumar, V., Nilsson, M. A. & Janke, A. Whole-genome sequencing of the blue whale and other rorquals finds signatures for introgressive gene flow. *Sci. Adv.* **4,** eaap9873 (2018).
